# Expression of PD-L1 in pleural effusion of advanced lung adenocarcinoma and its relationship with DNA ploidy

**DOI:** 10.12669/pjms.41.11.11655

**Published:** 2025-11

**Authors:** Juan Wu, Liangyan Huang, Ying Liu, Rui Wang, Yun Du

**Affiliations:** 1 Juan Wu, The Cancer Center, The Fourth Hospital of Hebei Medical University, Shijiazhuang 050000, Hebei, China; 2 Liangyan Huang, The Cancer Center, The Fourth Hospital of Hebei Medical University, Shijiazhuang 050000, Hebei, China; 3 Ying Liu, The Cancer Center, The Fourth Hospital of Hebei Medical University, Shijiazhuang 050000, Hebei, China; 4 Rui Wang, The Cancer Center, The Fourth Hospital of Hebei Medical University, Shijiazhuang 050000, Hebei, China; 5 Yun Du, The Cancer Center, The Fourth Hospital of Hebei Medical University, Shijiazhuang 050000, Hebei, China

**Keywords:** Pleural effusion, Lung adenocarcinoma, Programmed death-ligand 1, DNA aneuploidy

## Abstract

**Objective:**

Programmed death receptor-1 (PD-1) negatively regulates antigen receptor signaling upon binding to its ligands, programmed death-ligand 1 or 2 (PD-L1/2), enabling tumor cells to evade immune surveillance. This study aimed to investigate the expression of PD-L1 in tumor cells present in pleural effusion (PE) from lung adenocarcinoma (LUAD) with metastasis and its relationship with DNA ploidy. Furthermore, it sought to assess whether high PD-L1 expression enhances tumor cell proliferation and invasiveness.

**Methodology:**

This was a retrospective study. PE specimens were collected from patients diagnosed with stage IV LUAD, meeting the malignant tumor UICC (Union for International Cancer Control) TNM staging system, at the Fourth Hospital of Hebei Medical University and Hebei Tumor Hospital between July 2021 to December 2023. The expression of PD-L1 in LUAD cells in PE was detected using immunocytochemistry, and the invasiveness of tumor cells with varying PD-L1 expression levels was evaluated through DNA quantitative analysis.

**Results:**

The positive expression rate of PD-L1 in PE from advanced LUAD was 60.22%. Variations in PD-L1 expression were associated with the mean DNA index values exceeding 2.5 among the top 20 tumor cells analyzed.

**Conclusion:**

Compared with the non-expression group, the low-expression PD-L1 group demonstrated greater tumor cell invasiveness.

## INTRODUCTION

Lung cancer ranks first among newly diagnosed cancer cases worldwide, accounting for 11.4% of the global total, and is a leading cause of cancer-related deaths, contributing to 18.0% of all cancer fatalities.^1^,^2^ Since early-stage lung cancer often lacks significant symptoms, many patients are diagnosed at an advanced stage when they develop symptoms, with some presenting pleural effusion (PE) as the initial manifestation, thereby losing the opportunity for surgical resection.

In recent years, advancements in understanding tumor immune evasion mechanisms have positioned immunotherapy as a novel treatment paradigm for non-small cell lung cancer. Programmed death receptor-1 (PD-1) is a receptor primarily expressed in T cells, while its ligands, programmed death-ligand 1 (PD-L1) and programmed death-ligand 2 (PD-L2), are expressed in various cell types, including normal lymphocytes and diverse tumor cells such as lung cancer and melanoma. When PD-1 binds to its ligands PD-L1/2, it negatively regulates antigen receptor signaling, suppresses T-cell proliferation, and reduces cytokine production, enabling tumor cells to evade T-cell-mediated immune surveillance.^3^ Cells expressing PD-L1 are believed to exhibit high proliferative activity.^4^

However, whether this high proliferative activity contributes to increased tumor cell invasiveness and the development of malignant PE remains to be further explored. DNA aneuploidy, indicative of chromosomal instability in cells, represents one of the most common genetic abnormalities associated with tumorigenesis and progression. Tumor cells with high aneuploidy are considered to have a higher propensity for vascular invasion and lymph node metastasis. DNA aneuploidy, as a molecular indicator, can be employed to evaluate the invasiveness of tumor cells in PE with varying levels of PD-L1 expression. This may aid in identifying subgroups of patients with more invasive tumors.^5^-^9^

Several studies have confirmed that PD-L1 expression in cytological specimens from PE is highly consistent with that in lung tissue specimens, accurately reflecting the histological expression at the primary lung site.^10^ This study used immunocytochemical methods to detect PD-L1 expression in lung adenocarcinoma (LUAD) cells within PE, analyze the relationship between PD-L1 expression and DNA ploidy, and assess whether high PD-L1 expression enhances tumor cell proliferation and invasiveness.

## METHODOLOGY

This was a retrospective study. PE specimens were collected from patients diagnosed with stage IV LUAD, meeting the malignant tumor UICC (Union for International Cancer Control) TNM staging system, at the Fourth Hospital of Hebei Medical University and Hebei Tumor Hospital between July 2021 to December 2023.

### Ethical Approval:

The study was approved by the Institutional Ethics Committee of The Fourth Hospital of Hebei Medical University (No.: 20220918; Date: December 1, 2022), and written informed consent was obtained from all participants.

### Inclusion criteria:

- The PE submitted for examination must contain sufficient tumor cells for routine cytology, immunocytochemistry and DNA testing.
- Patients must have no other tumors.
- Patients must have no immune system diseases and must not have received any immunosuppressive therapy prior to diagnosis.

### Exclusion criteria:

- Patients who cannot evaluate the primary lesion after undergoing surgical treatment.
- Patients with missing or unclear case data.
- Patients who have received immunosuppressive therapy before being sent for pleural effusion testing.

### Immunocytochemical staining of tumor cell PD-L1 and interpretation:

A disposable membrane filter cell collector was used to filter portions of the PE, capturing and enriching tumor cells. The enriched cells were evenly smeared onto glass slides and fixed with a 95% ethanol solution. The remaining PE was used to prepare cell paraffin blocks. All PE samples underwent routine cytological diagnosis and immunocytochemical staining for markers such as thyroid transcription factor 1 (TTF-1), Napsin A, p40, p63, Wilms’ tumor 1 (WT-1), calretinin, synaptophysin (Syn), and carcinoembryonic antigen (CEA). Each case was independently diagnosed by two experienced cytopathologists as metastatic LUAD in PE. PD-L1 results interpretation: PD-L1 expression was detected using the Roche Ventana SP263 rabbit monoclonal antibody on a Roche fully-automated immunohistochemistry system. TTF-1, Napsin A, and PD-L1 were all tested on the same cell paraffin block to eliminate the potential influence of nonspecific expression across different blocks. PD-L1 expression was evaluated using the tumor proportion score (TPS), which was defined as the percentage of tumor cells in the sample showing partial or complete membrane staining at any intensity. The results were categorized as follows: (1) TPS < 1%: No expression; (2) TPS 1%-49%: Low expression; (3) TPS ≥ 50%: High expression.

### DNA quantitative analysis:

The DNA index (DI) represents the DNA content and is calculated as follows: DI = IOD of the measured cells / Average IOD of normal cells, wherein integrated optical density is abbreviated as IOD. When a measured cell is in the G0/G1 phase, its IOD is nearly identical to the average IOD of normal cells, and the DI value is considered 1 (i.e., 2C). In the G2/M phase, the cell’s IOD is approximately twice the average IOD of normal cells, yielding a DI value of 2 (i.e., 4C). If there were more than three cells with a DI > 2.5 (5C; aneuploid cells) or the proportion of cells with DI ≥ 2 among the total analyzed cells reached 10% and above, the PE samples were deemed to test positive for DNA aneuploidy. The fully automated LD DNA-ICM system displays the total number of epithelial cells in PE specimens, the number of cells with DI > 2.5, the DI values of the top 20 cells with the highest DNA content, and the number of aneuploid peaks.

### Statistical analysis:

Data were processed using the SPSS 26.0 software. The consistency between cytological and histological PD-L1 TPS was assessed using the Kappa coefficient. The relationship between DNA aneuploidy and PD-L1 expression was analyzed using the Kruskal-Wallis H test. A P-value <0.05 was considered statistically significant.

## RESULTS

A total of 93 patients with LUAD and PE metastasis were included in this study, comprising 42 males and 51 females, with a median age of 63 years. The PD-L1 TPS in PE specimens was distributed as follows: TPS < 1%: 37 cases (39.78%); TPS 1%-49%: 35 cases (37.63%); TPS ≥ 50%: 21 cases (22.58%). The overall PD-L1 positive expression rate in PE specimens was 60.22%. PD-L1 detection was also performed on 63 cases with corresponding histological specimens. The distribution was as follows: TPS < 1%: 27 cases; TPS 1%-49%: 27 cases; TPS ≥ 50%: 9 cases. The concordance rate between PE and histological specimens was 77.8%, with no statistically significant differences between the two groups. PD-L1 expression was consistent between the two specimen types (Kappa = 0.650, P < 0.01).

The maximum DI value (P = 0.446) and the presence of aneuploid peaks (P = 0.068) did not show significant differences across the PD-L1 expression subgroups (P > 0.05). However, the average DI value for the top 20 cells with DI > 2.5 in the low PD-L1 expression group (1%-49%) was significantly higher than that in the high- and non-expression groups (P = 0.031) (Table-I). Pairwise comparisons revealed that the number of aneuploid cells increased significantly when comparing the non-expression group (TPS < 1%) with the low-expression group (TPS 1%-49%) (P = 0.028) (Table-II).

**Table-I:** Relationship between PD-L1 expression and DNA aneuploidy.

PD-L1 expression	DI-MAX			Average DI (Top 20 cells with DI > 2.5)		
	Mean rank	χ²	P value	Mean rank	χ²	P value
<1%	43.35	1.615	0.446	38.26	6.980	0.031*
1%-49%	53.17			54.81		
≥50%	46.14			49.38		

***Note:***

* P < 0.05 indicates a difference of statistical significance.

**Table-II:** Pairwise comparison of the average DI value for the top 20 cells with DI > 2.5 across different PD-L1 expression groups.

PD-L1 between pairs	P value	Adjusted P value
<1% vs. ≥50%	0.131	0.394
<1% vs. 1%-49%	0.009	0.028*
1%-49% vs. ≥50%	0.466	1.000

***Note:***

* P < 0.05 indicates a difference of statistical significance.

## DISCUSSION

The biological behavior of LUAD under different PD-L1 expression states remains unclear. Some studies suggest that tumor cells with high PD-L1 expression exhibit greater proliferative activity, as indicated by higher Ki-67 indices,^11^ and may exhibit epithelial-to-mesenchymal transition and cancer stem cell-like characteristics, conferring stronger invasiveness and facilitating metastasis to the pleural cavity, leading to malignant effusion.^12^ This study utilized an objective, molecular-based indicator to evaluate the impact of PD-L1 expression on the biological behavior of LUAD. Numerous studies^13^-^20^ have established that DNA aneuploidy reflects chromosomal instability, one of the most common genetic abnormalities associated with tumor development and progression. Maounis et al.^21^ reported that DNA ploidy, determined via image analysis, could identify patient subgroups with more aggressive tumors.

Other studies^22^,^23^ link high DNA aneuploidy content with increased vascular invasion and lymph node metastasis. Therefore, DNA aneuploidy may serve as an objective genetic marker to assess LUAD’s biological behavior across different PD-L1 expression states. Our results revealed that, compared with the non-expression group, the low PD-L1 expression group exhibited higher DI values and a greater number of aneuploid cells, indicating greater genetic instability and malignancy, consistent with prior studies.^24^ Based on these findings, we hypothesize that advanced LUAD with low PD-L1 expression is more invasive than non-expressing LUAD.

No significant differences in DNA aneuploidy were observed between the high PD-L1 and low PD-L1 expression groups. This suggests that the biological behavior of the low PD-L1 expression group may differ from the high-expression group. PD-L1 may affect tumor-infiltrating lymphocytes through various mechanisms and is expressed in a variety of immune cells. Studies report that PD-L1 expressed in tumor-associated macrophages and tumor-associated neutrophils suppress tumor-infiltrating lymphocytes within the tumor immune microenvironment.^25^,^26^ This indicates that tumor cells with differing PD-L1 expression may have distinct impacts on the tumor immune microenvironment, which warrants further investigation.

### Limitations

It includes a small number of samples. In view of this, more samples should be included in future studies to further validate the findings of this study.

## CONCLUSIONS

Tumor cells with low PD-L1 expression exhibit more aneuploid cells compared with non-expressing cells. This indicates greater invasiveness of those with low PD-L1 expression, suggesting a potential key mechanism responsible for serosal metastasis in patients.

### Authors’ Contributions:

**JW** and **LH:** Conceived, designed the study and Review.

**YL** and **RW:** Collected the data, critical revciew and performed the analysis.

**YD:** Collected the data, performed the analysis, critical review, were involved in the writing of the manuscript.

All authors have read and approved the final manuscript.
